# COVID-19 Pandemic and Reduced Physical Activity: Is There an Impact on Healthy and Asthmatic Children?

**DOI:** 10.3389/fped.2021.695703

**Published:** 2021-09-08

**Authors:** Giuliana Ferrante, Desiree Mollicone, Salvatore Cazzato, Enrico Lombardi, Massimo Pifferi, Attilio Turchetta, Giancarlo Tancredi, Stefania La Grutta

**Affiliations:** ^1^Department of Health Promotion, Mother, and Child Care, Internal Medicine and Medical Specialties, University of Palermo, Palermo, Italy; ^2^Department of Anatomical and Histological Sciences, Legal Medicine and Orthopedics, Sapienza University of Rome, Rome, Italy; ^3^Department of Mother and Child Health, Salesi Children's Hospital, Ancona, Italy; ^4^Pediatric Pulmonary Unit, Meyer Pediatric University Hospital, Florence, Italy; ^5^Department of Paediatrics, University Hospital of Pisa, Pisa, Italy; ^6^Sport Medicine Unit, Bambino Gesù Children's Hospital, Rome, Italy; ^7^Pediatric Department, Sapienza University of Rome, Rome, Italy; ^8^National Research Council of Italy, Institute for Biomedical Research and Innovation (IRIB), Palermo, Italy

**Keywords:** asthma, children, COVID-19, physical activity, sedentary behavior

## Abstract

Physical activity (PA) has been seen to improve asthma symptoms, lung function, and quality of life, as well as to reduce airway inflammation and bronchial responsiveness. As a consequence of the COVID-19 pandemic, the minimal amount of PA recommended by the World Health Organization—i.e., about 60 min/day of moderate-to-high intensity—is difficult to achieve for many children, particularly those living in urban areas. Short-term changes in PA because of the COVID-19 pandemic may become habitual, increasing the risk of adverse asthma outcomes in children. Indeed, prolonged home confinement during the COVID-19 pandemic reduces PA levels and increases sedentary behaviors, possibly impairing immune system function and increasing susceptibility to inflammatory diseases. However, there is limited evidence regarding the effects of lockdown due to COVID-19 on PA and sedentary behaviors in asthmatic children. Given that children stay longer indoors, indoor air pollution represents a major issue to consider during home confinement. This narrative review aims to summarize the available evidence about the impact of decreased PA and increased sedentary behaviors on children with asthma during the COVID-19 pandemic. In addition, strategies for supporting PA in children with asthma during the COVID-19 pandemic are suggested, also looking at the issue of indoor air quality.

## Introduction

In December 2019, a public health emergency started due to an outbreak of novel coronavirus, now referred to as severe acute respiratory syndrome coronavirus 2 (SARS-CoV-2), in Wuhan, China (1). SARS-CoV-2 rapidly spread worldwide with high morbidity and mortality. On March 11, 2020, a global pandemic was declared by the World Health Organization (WHO). Government measures to counteract diffusion of the virus consist in hand and respiratory hygiene, physical distancing, remote working, home-quarantine and lock-down including closure of schools and gyms and limitation of recreational activities. During home-quarantine, ordinary daily routine has been radically modified with increasing risk of unhealthy habits (2). In particular, a general decline in physical activity (PA) has been reported. Indeed, social distancing and the obligation to stay at home potentially decrease opportunities for PA. The WHO 2020 guidelines on PA and sedentary behavior advised that children and adolescents should have as a minimum about 60 min/day of moderate-to-vigorous mostly aerobic PA, across the week. Moreover, they should limit the amount of sedentary time, particularly the amount of time watching TV and playing videogames (3). Nonetheless, due to the COVID-19 pandemic, the minimum recommended by WHO is difficult to achieve for many children, particularly those who live in urban areas (4).

Therefore, the “inactivity pandemic” emerged as one of the most relevant adverse effects due to the long period of home-quarantine (5) and social isolation with potential detrimental consequences on physical well-being of children, particularly those with chronic diseases, disabilities and special health needs (6, 7).

Regular PA is recognized to be a good way to avoid diseases and keep healthy (8). In particular, there is evidence that training programs are beneficial in combating chronic respiratory diseases like asthma (9). It has been shown to improve asthma symptoms, lung function, and quality of life, and to reduce airway inflammation and bronchial responsiveness (10–12). Moreover, PA is able to modulate the immune system function and can be protective against overweight and obesity, promoting a reduction of systemic inflammation (13).

Previous studies evaluated the effects of PA on childhood asthma in order to define its potential benefits. Systematic reviews have suggested that physical training (including running, swimming, and walking) may reduce airway inflammation in asthmatics (14) and that physical training (including running, gymnastics, cycling, swimming, weights and walking) has some positive effects on quality of life, without significantly improvement of resting lung function (15). A systematic review reported that exercise training (defined as training for ≥7 days, ≥2 times per week, ≥5 training sessions in total) improved asthma symptoms, quality of life, exercise capacity, and lung function in adults and children (11). A more recent systematic review demonstrated that aerobic PA (any type of physical exercise training lasting at least 20 min per day, undertaken at least two times per week for a minimum duration of 4 weeks) improves asthma in children and adults by reducing the prevalence and frequency of nocturnal symptoms (16). In particular, among the studies performed on children, only one reported significant improvement in the nocturnal symptoms scores in both the groups experiencing two modalities of aerobic exercise (active video game and treadmill) for 8 weeks (*p* = 0.1) (17).

Worldwide, an alarming and increasing number of people are not sufficiently physically active; and it is feared that this number will increase in quarantine periods (18).

As decreased PA and increased sedentary behaviors during the quarantine period are expected to put a significant burden on children with chronic diseases, this narrative review aims to summarize the available evidence about the impact of decreased PA and increased sedentary behaviors on children with asthma during the COVID-19 pandemic. In addition, strategies for supporting PA in children with asthma during the COVID-19 pandemic also looking at the issue of indoor air quality.

## Methods

We examined original papers in English in the PubMed, Scopus and Embase databases using the following keywords, separately and in combination: asthma, COVID-19, physical activity, and children. The age range was birth to 18 years. There were no limitations regarding the date or country. We also delved into the WHO website and searched the reference lists of the retrieved articles.

## The COVID-19 Pandemic: Decreased Physical Activity and Increased Sedentary Behaviors in Normal Children

It is evident that during the COVID-19 pandemic, children and young people globally spent less time in PA. Reduced PA and increased sedentary behaviors are well-known risk factors for obesity and poor cardiorespiratory fitness among children, which can lead to serious health consequences. Moreover, PA is expected to have a beneficial impact on the immune system and the risk for upper respiratory tract infections (19). The current scenario of the COVID-19 pandemic undoubtedly represents an alarming situation for children, with effects on PA and sedentary behaviors, which were investigated by several studies (Table 1). Among studies conducted in Europe, one study in Italy examined the possibility that factors contributing to weight gain among children and adolescents with overweight and obesity were accentuated during lockdown. The authors found that time spent in sports activities decreased by 2.30 ± 4.60 h/week (*p* = 0.003) whereas screen time increased by 4.85 ± 2.40 h/day (*p* < 0.001). In addition, an inverse correlation was observed between changes in sport activities and changes in time spent in front of a screen, although at borderline significant level (20). A study conducted on Spanish pre-school children during the lockdown reported that PA decreased (mean difference, MD = −43.3 min per day) while sedentary time increased (MD = +50.2 min per day) (21). In addition to these data, an online survey administered in Spain to 516 parents serving to collect data about 860 pre-schoolers, children and adolescents showed a significant decrease in PA for all age subgroups before and during the confinement; in particular, the highest reduction of weekly minutes [−120.4 (SD 159.0)] was reported in children aged between 6 and 12 years. Also, screen time showed a significant increase for all participants and all age subgroups; the highest increase [+3.3 (SD 2.1) more daily hours] was observed for adolescents (13–16 years) (22). Conversely, Swedish pre-schoolers reported an increased in PA (MD = +53 min per day) during the lockdown, but also spent more time watching TV or playing videogames (MD = +30 min per day). These findings could be linked to the fact that pre-schools, playgrounds, and parks in Sweden remained open and organized sports and activities for children continued even during the current pandemic; at the same time, the increased screen time observed could be connected to the fact that children stayed at home if they were symptomatic, allowing them to spend more time than usual in front of screens (23).

**Table 1:** Effects of the COVID-19 pandemic on physical activity and sedentary behaviors in normal children.

**References**	**Country**	**Study type**	**Study population**	**Aim and study procedures**	**Results**	**Limitations**
**European studies**						
Pietrobelli et al. (20)	Italy	Longitudinal observational study (baseline assessment: May-June 2019; second evaluation: March-April 2020)	41 children (mean age 13.3.1 years)	To test the hypothesis that factors contributing to weight gain among children and adolescents with overweight and obesity are exacerbated during COVID-19 pandemic-associated lockdown by questionnaire	Time spent in sports activities decreased by 2.30 ± 4.60 h/week (*p* = 0.003); screen time increased by 4.85 ± 2.40 h/day (*p* < 0.001). An inverse correlation was observed between changes in sport activities and changes in time spent in front of a screen, although at borderline significant level (*r* = −0.27, borderline significant at *p* = 0.084)	Self-reported data, small sample size
Alonso-Martínez et al. (21)	Spain	Cross-sectional study (baseline assessment: September–December 2019; second evaluation: March–April 2020)	268 pre-schoolers aged 4–6 years	To examine the effects of the COVID-19 lockdown on PA, sedentary time, and sleep assessed using accelerometry in the week in which the Spanish national state of alarm was declared (*n* = 21)	Decrease in total PA [mean difference (MD) = 43.3 min per day, 95% CI 68.1–18.5], and an increase in sedentary time (MD = 50.2 min per day, 95% CI 17.1–83.3)	Small sample size with accelerometry data and a short time of monitoring
López-Bueno et al. (22)	Spain	Cross-sectional study conducted in March-May 2020	860 children and adolescents aged between 3 and 16 years (mean age 9.6 ± 3.9 years)	To investigate the impact of the COVID-19 confinement on health-related behaviors in children using an online survey administered to parents	Significant reduction of weekly minutes of PA during the confinement (−102.5, SD 159.6) (*p* <0.001) and increase of daily hours of screen exposure (2.9, SD 2.1) (*p* <0.001)	Self-reported data, wide age range, convenience sampling
Nyström et al. (23)	Sweden	Cross-sectional study (baseline assessment: March–May 2019; second evaluation: May–June 2020)	82 children (mean age 4 ± 0.5 years)	To assess how movement behaviors have been affected in pre-schoolers during the COVID-19 pandemic assessed using a questionnaire filled in by parents	PA, time spent outside on weekdays and weekend days, and screen time significantly increased (+53; +124; +68; +30 min/day, respectively, *p* <0.001).	Self-reported data by not validated questionnaire
**International studies**						
Sá et al. (24)	Brazil	Cross-sectional study conducted in March–April 2020	806 children aged 0 to 12 years	To evaluate how families with children aged <13 years faced the period of social isolation resulting from the COVID-19 pandemic, especially regarding the time spent on PA, games, outdoor activities and screen time by questionnaire	Significant reduction in the percentage of total PA time (percentage of reported hours: 26.11% in children aged 0–2 years; 19.56% in children aged 3–5 years; 10.99% in children aged 6-9 years; 9.77% in children aged 10–12 years) and an increase in total sedentary time (percentage of reported hours: 73.89% in children aged 0–2 years; 80.44% in children aged 3–5 years; 89.01% in children aged 6–9 years; 90.23%in children aged 10–12 years) (*p* <0.001)	Self-reported data
Dunton et al. (25)	U.S.	Prospective study conducted in April–May 2020	211 children (mean age 8.73 ± 2.58 years)	To examine the effects of the COVID-19 pandemic on PA and sedentary behavior in children using an online survey administered to parents	The most common physical activities during the early-COVID-19 period were free play/unstructured activity (e.g., running around, tag) (90%) and going for a walk (55%). Children engaged in about 90 min of school-related sitting and over 8 h of leisure-related sitting a day. Parents of older children (aged 9–13) vs. younger children (aged 5–8) were half as likely [OR = 0.54, 95% CI (0.31, 0.93)] to have a one-unit change in the perception their children had done less sedentary behavior in past 7 days as compared to February 2020.	Self-reported data, sample not representative compared to U.S. demographic data and not geographically equally distributed
Dayton et al. (26)	U.S.	Retrospective case-control study (first phase March 2020; second phase June 2020)	20 children and young adults (mean age cases: 15.2 ± 3.2; controls: 14.5 ± 3.2)	To evaluate the effect of deconditioning from social distancing and school shutdowns during the COVID-19 pandemic on the cardiovascular fitness of healthy children	The maximal oxygen uptake (VO2 max) in the post-COVID cohort was markedly lower than in the pre-COVID cohort (39.1 vs. 44.7, *p* = 0.031); the percentile of predicted VO2 max was significantly lower in the post-COVID cohort (95 vs. 105%, *p* = 0.042). There was a trend for the anaerobic threshold to be lower in the post-COVID cohort, even though it did not reach statistical significance (21.5 vs. 24.6, *p* = 0.082)	Small sample size, comparisons of exercise performance not obtained on the same patient pre- and post-COVID but through matched controls, different exercise protocols

Similar findings were obtained by international studies conducted in Brazil and U.S. An online survey launched in Brazil to identify the behavior of children under 13 years during confinement due to the COVID-19 pandemic, indicated a significant reduction in the percentage of total PA time (percentage of reported hours: 26.11% in children aged 0–2 years; 19.56% in children aged 3–5 years; 10.99% in children aged 6–9 years; 9.77% in children aged 10–12 years) and an increase in total sedentary time (percentage of reported hours: 73.89% in children aged 0–2 years; 80.44% in children aged 3–5 years; 89.01% in children aged 6–9 years; 90.23% in children aged 10–12 years) (*p* < 0.001) with age. 46.1% of parents reported that children were having much less PA, and 37% that PA was less frequent than during the school period; 38% reported that screen time was higher than in regular school hours, and 36.9% that it was much higher (24). According to an online survey conducted in the U.S., about one child in three had online school lessons early on after the COVID-19 outbreak. Moreover, parents of children aged 9–13 years noticed more marked decreases in PA and larger increases in sedentary behaviors compared with parents of younger children (aged 5–8 years). Overall, children were reported to spend most of the time watching television/videos/movies, doing school-related work, and playing video games. Interestingly, whereas school-related sedentary time accounted for roughly 90 min per day, sitting for leisure activities accounted for over 8 h per day. In particular, older children and girls generally spent more time in a sedentary state than younger children and boys, which implies that these groups of children may be at greater risk of unhealthy lifestyle (25). In a single-center, retrospective case-control study, 10 healthy children who underwent cardiopulmonary exercise testing were compared to a matched cohort before the COVID-19-related lockdown began. The maximal oxygen uptake (VO2 max) in the post-COVID cohort was markedly lower than in the pre-COVID cohort (39.1 vs. 44.7, *p* = 0.031) and the percentile of predicted VO2 max was significantly lower in the post-COVID cohort (95 vs. 105%, *p* = 0.042), indicating a significant decline in the physical fitness of healthy children due to the COVID-19 pandemic and restrictions linked to it (26).

Overall, the available evidence suggests a decrease in PA generally associated with increased time spent in front of a screen by children and adolescents during the present pandemic. However, it should be pointed out that several limitations in methodology affected the published results; therefore, the available findings should be interpreted with caution.

## Decreased Physical Activity and Increased Sedentary Behaviors in Asthmatic Children, and Its Impact During the COVID-19 Pandemic

In the period of the COVID-19 pandemic, an objective decrease in PA levels has been observed in children with asthma. Using wearable sensors to continuously track personal location and PA, Kouis et al. assessed changes in mobility of asthmatic children in Cyprus and Greece, reporting an overall increase of time spent at home and a decrease of PA level (27). In Israel, a study using an electronic questionnaire submitted during lockdown (March–May 2020) to caregivers of children and adolescents with asthma and other chronic respiratory disorders demonstrated that patients aged >5 years had increased screen time, and decreased PA compared to their younger counterparts (*p* = 0.008 and *p* < 0.001, respectively) (28).

Studies investigating PA in childhood asthma showed that children with asthma tended to be less physically active than their healthy peers. Groth et al. found that adolescents with asthma in the U.S. had less PA than those without asthma. In particular, boys with asthma were less physically active than boys without asthma (high-intensity exercise: MD – 0.81, *p* < 0.05) and girls were less active than boys regardless of asthma status (high-intensity exercise: MD – 0.72, *p* < 0.001). In addition, gender was found to be associated with sedentary behavior: screen time was higher in boys than girls (hours/day use pc or watch video games: MD: −0.32, *p* < 0.001) (29). Similarly, a study in Taiwan showed that children with moderate-to-severe asthma were less active than healthy children the same age. A correlation was found between PA and asthma severity level: children who had moderate or severe persistent asthma were more likely to be inactive (30).

These findings have not been confirmed by more recent studies, which showed similar levels of PA in children with asthma and healthy children. Within the UK Millennium Cohort, different levels of PA between children with asthma and controls were found but were not significant. However, it should be noted that in children with a recent asthma admission lower levels of total PA were observed than in controls. Overall, a low proportion (50–56%) of children, whether healthy or asthmatic, were found to comply with the daily recommended PA guidelines (31). A very recent systematic review of 16 studies did not report differences in PA levels for young people with asthma compared to healthy peers; nonetheless, PA levels remained generally inadequate in most studies (32). Another systematic review of 28 studies suggested that children and adolescents with asthma have similar moderate to vigorous PA, steps/day, and sedentary time compared to controls (33).

PA has been found to ensure better asthma control in children (34). A study on Brazilian schoolchildren found that those with controlled asthma were more active than those whose asthma was uncontrolled (*p* = 0.032). Moreover, in more active schoolchildren asthma was more likely to be controlled (OR = 1.5; 95%CI: 1.04–2.25) (35). Within the National Youth Fitness Survey, decreased aerobic fitness and increased sedentary time led to worse asthma outcomes including asthma attacks, wheeze with exercise, and wheeze with activity limitations, in children with doctor-diagnosed asthma (36). The effect of sedentary behaviors on childhood asthma outcomes needs to be further elucidated. Since sedentary children were found to present predominance of Th1 response in relation to active children, who instead showed higher levels of Interleukin 10 and higher regulatory T cell percentage, it could be hypothesized that regular PA may help to foster an anti-inflammatory profile. Consequently, prolonged social isolation during the COVID-19 pandemic, limiting PA, may lead to an increase in adiposity and susceptibility to inflammatory diseases (37).

Nonetheless, during the COVID-19 pandemic improved childhood asthma outcomes were observed, namely reduced acute attacks, emergency department visits, and hospitalizations as well as improved scores in asthma control measures and lung function. In particular, improved or unchanged asthma control during pandemic was reported by 90.2% of the participants to a multi-national cohort study including 1,054 children with asthma and 505 non-asthmatic children aged 4–18 years from 15 countries. These findings might be due to reduced exposure to outdoor asthma triggers, such as seasonal allergens and air pollutants, and increased treatment adherence (38). Noteworthy, social distancing and facemasks have been proposed to reduce respiratory viral infection, leading to less hospital admission for asthma exacerbation in adults. Wearing facemasks has been proved to be protective against respiratory infections which are a common trigger of asthma exacerbation (39). Indeed, a large study in Hong Kong reported that the number of admissions for asthma exacerbation was reduced by 53.2% (95% CI 50.4–55.8%, *p* < 0.0001) in 2020 compared with past 5 years; admissions for asthma exacerbation decreased by 0.8% with every 1% increase in masking (95% CI 0.8–0.9%, *p* < 0.0001) (40). However, Sykes et al. observed that, despite a decreased number of exacerbation-related admissions, adult patients with asthma showed a subjective decline in disease control and reported a negative impact on their mental health. Among those with severe asthma, 34.1% reported their disease control to be worse and 18.3% reported the pandemic as having a negative effect on their mental health. The sedentary lifestyle during the COVID-19 pandemic could be associated with worse disease outcomes in patients with severe asthma. However, the apparent discordance between perceived disease control and reduction in exacerbations requiring hospitalization in adults with severe asthma needs further investigation (41).

Among several factors that may have an impact on asthma severity and control, it should be acknowledged the discontinuation of in-person visits and the difficulty to perform lung function tests during the COVID-19 pandemic. A large online survey demonstrated that COVID-19 significantly impacted pediatric asthma services: 39% ceased in-person visits, 47% stopped accepting new patients, and 75% limited patients' visits. Importantly, more than 90% of participating centers launched virtual online or telephone consultations, while 73% used a helpline to remotely assist their patients. Forty-eight percent of the participants considered virtual visits a suboptimal clinical encounter, whereas 42% of patients found them acceptable, or, even, as good as face-to-face visits (42). In the context of the current pandemic, we think that telemedicine may be helpful especially to patients with severe or uncontrolled asthma who might be at increased risk due to lack of monitoring caused by social distancing and lockdowns. Taquechel et al. demonstrated an increased use of telemedicine and decreased overall asthma encounters during the COVID-19 pandemic (43). Indeed, the current pandemic has accelerated the adoption of telemedicine in healthcare, which however represents a new and understudied care model. Overall, future research will be essential in order to implement technology-supported monitoring at home which should be targeted to the individual child and integrated to healthcare systems.

## Strategies for Supporting Physical Activity in Children With Asthma During the Pandemic

**Figure 1:**
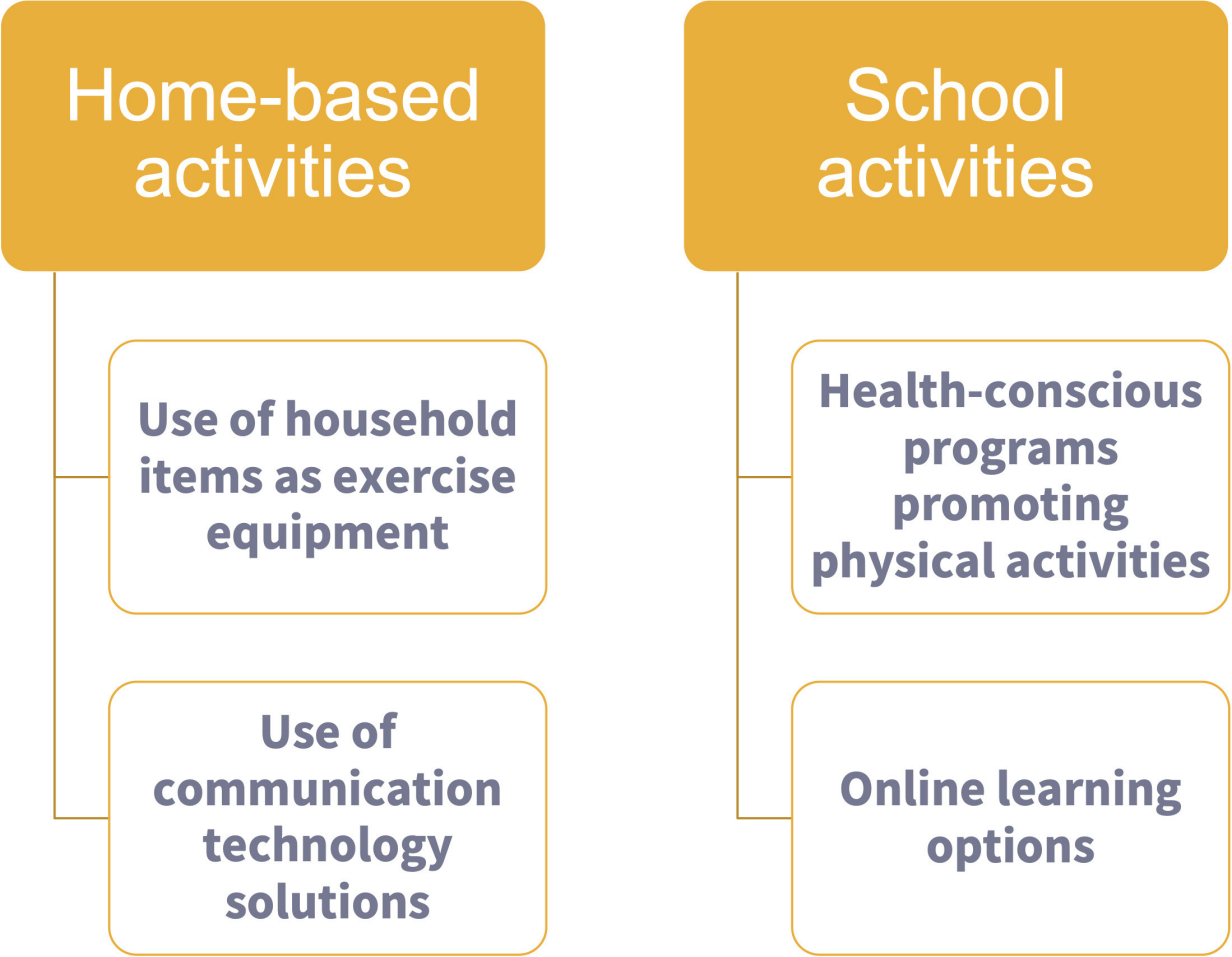
Strategies for encouraging physical activity in children during the pandemic at home and school.

Specific suggestions focused on decreasing sedentary behavior are vital, especially during a lockdown period, where it can be difficult to achieve moderate-vigorous PA (Figure 1). Promoting home-based activities that increase mobility can break sedentary behavior and increase levels of PA, thus enhancing health and well-being. Therefore, if the pandemic goes on, it will be fundamental to ensure that all children continue to have PA despite home confinement. Schools can integrate health-conscious programs by encouraging good personal hygiene, physical activities, appropriate diet, and good sleeping habits, in their curricula (44). For children having schooling online, it would be useful to provide opportunities for outdoor exercise without shareable equipment (45). However, measures that encourage and maintain regular PA in children cannot solely be dependent on school activities. Parents and caregivers should incorporate PA in children's daily home activities (including using electronic means to favor participation), but also the media and governments should provide regular messages promoting PA and healthy movement behaviors (46).

The recommendations provided so far for maintaining an active lifestyle during the pandemic mainly target the general population, and not so much vulnerable individuals like children with asthma (47, 48). Therefore, personalized PA programs with supervision are urgently required, possibly to be delivered and disseminated through communication technology solutions. Indeed, in the times of the pandemic, the use of digital tools to support and maintain PA could be particularly useful in children with chronic diseases (49). Recently, “serious games” (SGs), which are games not primarily oriented toward entertainment, enjoyment or fun, have provided new opportunities for constructive learning and training (50, 51). A very recent systematic review did not evidence that SGs improve PA levels in children with chronic diseases, likely due to small sample sizes and marked heterogeneity in study designs. Therefore, future research is required to evaluate the effectiveness of SGs in enhancing PA and healthy movement behaviors, especially in children with asthma (52). In a context of social distancing, Active video games (AVGs) can be an interesting alternative to increase PA levels in children (53). A controlled trial on children with moderate to severe asthma who were randomly allocated to either a video game group (VGG) or a treadmill group (TG) in an 8-week program with 2 weekly 40-min sessions found improvements in both groups with regard to asthma control and capacity. However, a marked reduction in exhaled nitric oxide levels was found in the VGG group [23.3 ± 10.9 ppb post-VGG vs. 35.5 ± 19.7 ppb pre-VGG; ΔFeNO −13.2 (8.22) ppb, *p* < 0.05], as well as a higher maximum energy expenditure (7.31 ± 1.64 cal/min in VGG vs. 5.68 ± 1.27 cal/min in TG, *p* < 0.05), suggesting that aerobic training linked to active videogames had a beneficial impact on asthma control, exercise capacity and airway inflammation (17). More recently, a cross-sectional study on children submitted to a cardiopulmonary exercise test reported that active videogames can promote intense physical exercise and can be considered a motivating and efficient treatment modality for children with asthma (54).

In the pandemic period, children may experience negative feelings promoted by social isolation, which in turn may have an impact on their health. Indeed, a link between psychosocial factors and asthma outcomes including symptoms and lung function has been previously demonstrated in young people (55). Therefore, it could be speculated that the use of AVGs implementing PA and exercise with digital games may positively impact psychosocial variables and health (56). Obviously, recommendations for promoting healthy movement behaviors during the COVID-19 pandemic should consider equity and minimal equipment, especially for children living in communities with little access to the internet (46).

## Performing Physical Activity at Home During the COVID-19 Pandemic: the Issue of Indoor Air Quality

As a consequence of governmental restrictions across the world due to the pandemic, time spent indoors is growing; therefore, exposure to certain indoor pollutants, such as tobacco smoke and other combustion products, has dramatically increased (57, 58). Indeed, homes often afford little space in relation to the number of inhabitants and often do not have adequate systems for ventilation and air renewal (59). In addition, human activities such as cooking (especially frying) and the movement of people in the room are significant contributors to concentrations of indoor pollutants (60). In particular, during aerobic exercise, inhaled air predominantly enters through the mouth, and respiratory uptake of airborne contaminants increases, with greater penetration to the lower lung regions. Furthermore, there is an association between the number of exercising individuals in confined environments and concentrations of coarse resuspended aerosols (PM_10−2.5_) (61). In addition, during the COVID-19 pandemic the increasing use of household cleaning products and disinfectants to reduce the potential of viral infection represented a particularly significant source of indoor pollution. As a consequence, a study in Spain reported that mean daily Volatile Organic Compound concentration rose by 37–559%, although this finding needs further research to quantify its actual impact on health (59). The increased intensity of time spent at home generates rising pollutant levels which are not always counteracted by ventilation in the home environment, due to its reduced effectiveness and/or to voluntary limitations. Overall, these factors may produce adverse effects on respiratory health, especially in vulnerable individuals. Indeed, increasing evidence has shown that the exposure of children to environmental indoor stressors is associated with respiratory conditions including wheezing, asthma, and rhinitis (62). It should also be pointed out that co-exposure to indoor allergens may aggravate airway inflammation, resulting in worsening of symptoms in sensitized children (63). The multifactorial routes of exposure to indoor pollutants should therefore be considered, also with a view to future confinement episodes for possible new waves of SARS-CoV-2.

**Figure 2:**
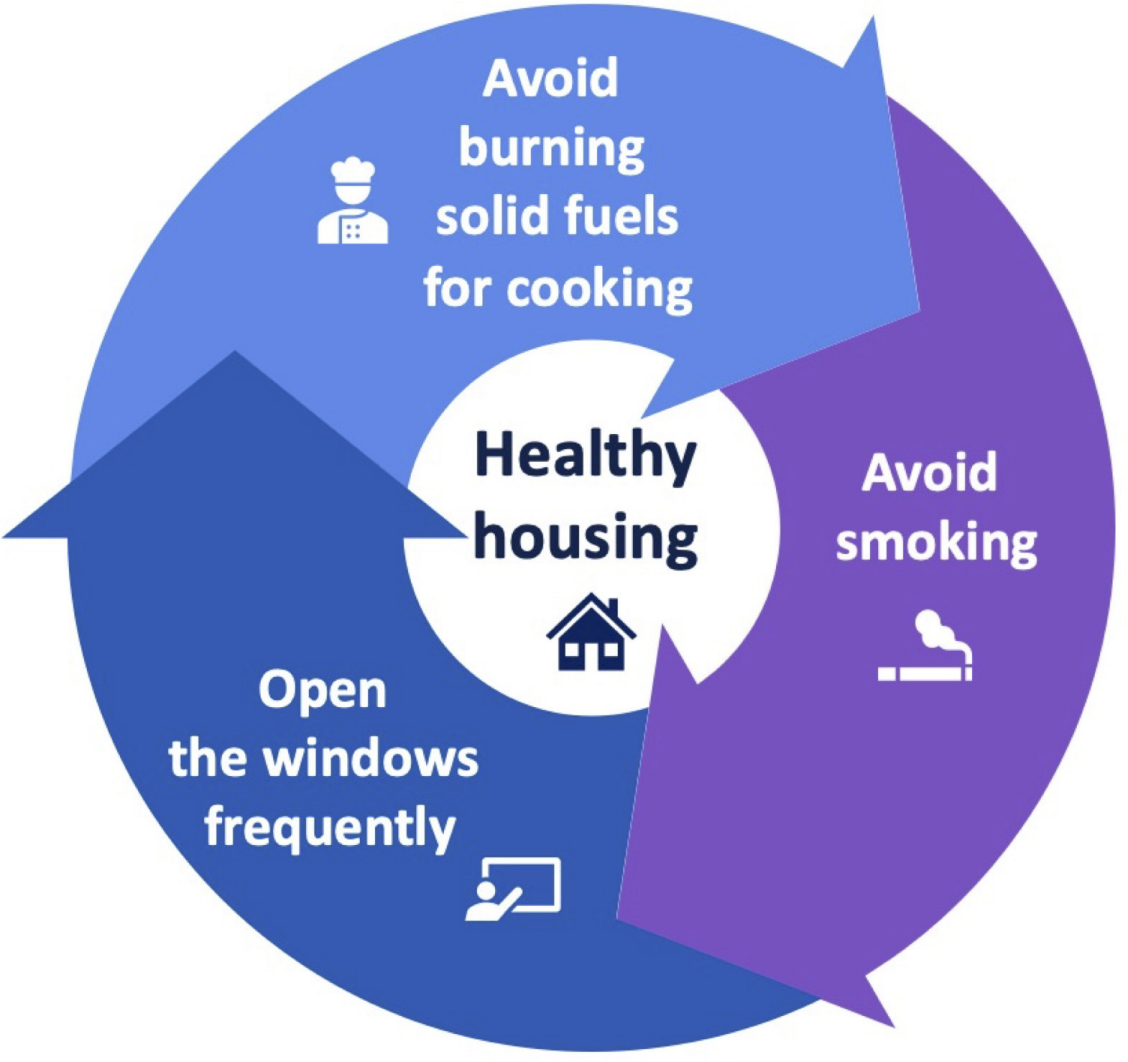
Main suggestions for “healthy housing.”

Some suggestions to improve indoor air quality may include the following: cooking in a healthier way in order to emit less pollutants; avoiding burning solid fuels; opening the windows frequently and using a kitchen ventilator when cooking, if possible; refraining from smoking at home (64); enhancing awareness about indoor air quality; and implementing educational programs at the community level (Figure 2). Increasing awareness about “healthy housing” may translate into preventive action by incorporating best practices to reduce the burden of indoor exposures on health, especially in vulnerable populations. Rapid developments in the scientific field of housing technology will likely help to achieve better indoor air quality in the near future to ensure safer indoor living conditions (65).

## Conclusions

The available evidence suggests a decrease in PA generally associated with increased time spent in front of a screen in both healthy children and adolescents during the pandemic. Decreased PA levels and increased sedentary behaviors have also been observed in children with asthma. Promoting home-based activities that increase mobility can lessen sedentary behavior and increase levels of PA, thus improving asthma outcomes in children. Therefore, if the pandemic goes on, it will be vital to ensure that children with asthma continue engaging in PA despite home confinement. Parents and caregivers should make PA a part of children's daily routine at home, but the media and governments should also provide regular messages to promote PA and healthy movement behaviors. Personalized PA programs with supervision are required, possibly to be delivered through communication technology solutions. Indeed, in these pandemic times, the use of digital tools to support and maintain PA could be particularly useful in children with chronic diseases. Improving indoor air quality is crucial to ensure a safe environment where children with asthma can perform PA.

## Author Contributions

DM, GF, GT, and SL: conceptualization, methodology, and writing original draft. GF and SL: review and editing. AT, GT, EL, MP, and SC: supervision. All authors have read and agreed to the published version of the manuscript.

## Conflict of Interest

The authors declare that the research was conducted in the absence of any commercial or financial relationships that could be construed as a potential conflict of interest.

## Publisher's Note

All claims expressed in this article are solely those of the authors and do not necessarily represent those of their affiliated organizations, or those of the publisher, the editors and the reviewers. Any product that may be evaluated in this article, or claim that may be made by its manufacturer, is not guaranteed or endorsed by the publisher.
